# Serum biomarkers for the prediction and diagnosis of preeclampsia: a systematic meta-analysis of diagnostic accuracy

**DOI:** 10.3389/fmed.2026.1861345

**Published:** 2026-08-03

**Authors:** Xiang Li

**Affiliations:** Department of Gynecology and Obstetrics, Tianjin Medical University General Hospital, Tianjin, China

**Keywords:** meta-analysis, prediction, preeclampsia, serum biomarkers, sFlt-1/PIGF

## Abstract

**Background:**

Preeclampsia, a life-threatening gestational complication for maternal-fetal health, necessitates prediction (across all trimesters) to improve clinical outcomes. Serum biomarkers, by revealing core pathological mechanisms such as angiogenic imbalance, have emerged as potential screening tools. However, the heterogeneity in their predictive efficacy and clinical utility requires evidence-based synthesis.

**Objective:**

To comprehensively evaluate the predictive potential of seven categories of serum biomarkers and clarify their clinical application value.

**Methods:**

Databases including PubMed, Embase, Cochrane Library, and Web of Science were searched from inception to January 20, 2026, to identify studies evaluating the diagnostic performance of serum biomarkers for preeclampsia. The methodological quality of included studies was assessed using the QUADAS-2 tool. Pooled sensitivity, specificity, positive likelihood ratio (PLR), negative likelihood ratio (NLR), diagnostic odds ratio (DOR), and area under the curve (AUC) were calculated using bivariate random-effects models and the hierarchical summary receiver operating characteristic (HSROC) model.

**Results:**

A total of 22 studies (7,772 participants) were included. The sFlt-1/PIGF ratio demonstrated the highest diagnostic accuracy, with a pooled sensitivity of 85%, specificity of 83%, and AUC of 0.923. For individual biomarkers, sFlt-1 showed a sensitivity of 78% (95% CI: 74–82%) and specificity of 79% (95% CI: 75–83%) with an AUC of 0.881; PIGF showed a sensitivity of 74% (95% CI: 70–78%) and specificity of 76% (95% CI: 72–80%) with an AUC of 0.859. Endoglin and leptin exhibited moderate diagnostic performance (AUCs of 0.814 and 0.768, respectively) but were limited by high heterogeneity (*I*^2^ > 75%). Activin A and inhibin A showed preliminary diagnostic potential (AUCs of 0.785 and 0.796, respectively), but these findings are based on only three studies each and require validation in larger prospective cohorts. Subgroup analysis revealed that diagnostic accuracy varied by gestational trimester, with the sFlt-1/PIGF ratio achieving the highest AUC (0.94) in the second trimester. Sensitivity analyses confirmed the robustness of the pooled estimates, and publication bias was assessed using Deeks’ funnel plot.

**Conclusion:**

This meta-analysis demonstrates that the sFlt-1/PIGF ratio exhibits superior diagnostic accuracy for preeclampsia, particularly during the second trimester.

## Introduction

Preeclampsia (PE), one of the most severe complications associated with hypertensive disorders during pregnancy, affects approximately 2-8% of pregnant women worldwide (1). Characterized by hypertension, proteinuria, maternal organ dysfunction, or utero-placental insufficiency, PE may lead to maternal and fetal mortality or other critical complications in severe cases (2). With the increasing prevalence of high-risk pregnancies globally, the incidence of PE has shown a rising trend. Consequently, achieving reliable risk assessment and accurate diagnosis of PE has emerged as a pivotal focus in obstetrical clinical practice and research. It is essential to emphasize that accurate identification of high-risk PE populations can facilitate the use of low-dose aspirin for the prevention of its onset (3). Elevated blood pressure, though a hallmark of PE, typically manifests after disease onset and lacks specificity (4). Proteinuria, another key indicator, may be absent in mild cases, leading to underdiagnosis or misdiagnosis (5). Existing screening criteria and predictive models predominantly depend on clinical features (e.g., prior obstetric history, family history) and fail to achieve precise individualized prediction. Thus, identifying biomarkers with higher sensitivity and specificity for risk stratification and diagnosis has become a critical research priority.

In recent years, circulating proteins and other serum-based molecular indicators (e.g., angiogenic factors, adipokines, and placental peptides) have gained prominence as non-invasive and convenient tools for risk assessment in the prevention and management of obstetric disorders (6). Compared to traditional methods, these circulating biomarkers offer predictive signals, reveal biological characteristics linked to PE pathogenesis, and demonstrate strong clinical potential due to their accessibility and quantifiability (7). Multiple studies have identified specific serum biomarkers strongly associated with PE development, including but not limited to placental growth factor (PIGF) and soluble fms-like tyrosine kinase-1 (sFlt-1) (3, 8). These biomarkers not only facilitate effective screening but also predict disease severity, progression, and clinical outcomes. Notably, the combined use of biomarkers significantly enhances the sensitivity and specificity of diagnosis, providing a scientific basis for clinical intervention (7). Despite the promise of serum biomarkers in PE prediction, research outcomes remain controversial. Variations in predictive performance across populations and environments, coupled with challenges in detection protocols, standardization, and cost-effectiveness, necessitate further optimization (9, 10). Therefore, a comprehensive analysis of the clinical utility of diverse serum biomarkers and an evaluation of their efficacy in risk assessment are imperative. This study conducts a systematic meta-analysis to synthesize and assess the magnitude of differences in biomarker levels (standardized mean difference, SMD) and the integrated application of serum biomarkers, aiming to optimize clinical screening strategies, promote their adoption in early PE diagnosis, and refine screening protocols. The ultimate goal is to enable timely identification, effective intervention, and reduction of PE incidence and associated complications.

## Method

### PICOS framework

The literature search and inclusion criteria were structured according to the PICOS framework:

Population (P): Pregnant women diagnosed with preeclampsia according to ACOG, ISSHP, or WHO criteria.
Intervention (I): Serum biomarker screening (single or combined), including but not limited to sFlt-1, PlGF, sFlt-1/PlGF ratio, endoglin, leptin, activin A, and inhibin A.
Comparison (C): Healthy pregnant women without preeclampsia.
Outcome (O): Diagnostic accuracy metrics, including sensitivity, specificity, positive and negative likelihood ratios, diagnostic odds ratio, and area under the curve (AUC).
Study design (S): Comparative trials, observational studies, and controlled clinical trials that reported sufficient data to construct 2 × 2 contingency tables.

### Literature search strategy

English databases including PubMed, Web of Science, Embase, and Cochrane Library were systematically searched from their inception through January 20, 2026. The structured search methodology integrated controlled vocabulary from Medical Subject Headings (MeSH) with natural language descriptors. Both MeSH terms and free-text words were combined using Boolean operators. Detailed search strategies for each database, including the specific search strings and filters applied, are provided in Supplementary Table 1. Core search terms included: (“preeclampsia” OR “pre-eclampsia” OR “hypertensive disorders of pregnancy”) AND (“biomarker” OR “serum marker” OR “sFlt-1” OR “PIGF” OR “endoglin” OR “leptin” OR “activin A” OR “inhibin A”) AND (“diagnosis” OR “prediction” OR “screening”).

Following the initial search, duplicates were removed. Two independent reviewers performed title and abstract screening, followed by full-text evaluation. Any disagreements were resolved through discussion or consultation with a third reviewer. A supplementary handsearching protocol was implemented to systematically scan reference sections of eligible articles, capturing potentially eligible studies not captured by database searches.

### Inclusion and exclusion criteria

Inclusion criteria: (1) Pregnant women diagnosed with preeclampsia (PE) according to established international clinical criteria (e.g., ACOG, ISSHP, or WHO). (2) Serum biomarker screening (single or combined profiles), including but not limited to sFlt-1, PlGF, sFlt-1/PlGF ratio, endoglin, leptin, activin A, and inhibin A. (3) Healthy pregnant women or non-preeclampsia comparison groups undergoing matching serum biomarker evaluation. (4) Comparative evaluation of diagnostic performance reporting raw data or comprehensive metrics [sensitivity, specificity, area under the curve (AUC), and likelihood ratios]. (5) Comparative trials, observational cohort studies, controlled clinical trials, and case-control studies evaluating biomarker levels between established PE cases and healthy controls. (6) Studies explicitly reporting the precise gestational week or trimester at the time of serum sample collection, categorized as: First trimester (<14 weeks) Second trimester (14–28 weeks) Third trimester (>28 weeks).

Exclusion criteria: (1) Non-preeclampsia populations or undiagnosed cases, or studies not utilizing serum-based biomarker evaluation (e.g., urine or tissue-only markers); (2) Basic science/*in vitro* research, animal models, case reports, conference abstracts, editorials, study protocols, and systematic reviews; (3) Studies with incomplete data, ambiguous gestational age reporting, unretrievable full texts, or duplicated/overlapping patient cohorts; (4) High-quality prospective prognostic studies (e.g., PROGNOSIS, PRAECIS, PETRA trials) that exclusively reported longitudinal, time-to-event outcomes (e.g., PPV/NPV within a specific 1-week or 4-week window) without extractable or calculable raw 2 × 2 contingency tables; (5) Articles published in languages other than English where an English full text was unavailable or translation support could not be reliably provided by the research team.

Methodological clarifications on study selection: (1) Justification for study design inclusion: Although the primary analytical objective of this review is prediction across trimesters, cross-sectional case-control designs comparing established PE cases with healthy controls were eligible, provided they reported sufficient raw data to populate the 2 × 2 contingency tables (true positives, false positives, true negatives, false negatives). These studies provide foundational data identifying biomarker signatures crucial for mathematical model development. (2) Justification for excluding landmark trials: Landmark prospective trials were systematically excluded because their time-dependent hazard ratios or windowed predictive metrics lacked the raw cross-sectional 2 × 2 data structures strictly required by our bivariate random-effects and HSROC meta-analytical models. (3) Potential limitations: The applied language restriction may introduce a localized selection bias, which is explicitly addressed as a methodological limitation in the Discussion section.

### Data extraction

Two investigators (XL and YZ) independently performed data extraction using a standardized form. Key elements included: bibliographic details (first author, publication year); study characteristics (design, sample size, geographical setting); participant demographics (mean age, gestational age); biomarker profiles (serum marker categories, detection methods); diagnostic accuracy metrics (sensitivity, specificity, AUC, true positives, false positives, true negatives, false negatives). Discrepancies were resolved by consensus or adjudication by a third reviewer (WC). A dual verification mechanism ensured data accuracy.

### Quality assessment

Methodological quality was assessed using the Quality Assessment of Diagnostic Accuracy Studies-2 (QUADAS-2) tool. Two independent reviewers (XL and YZ) evaluated each included study across four key domains: patient selection, index test, reference standard, and flow and timing. Specifically, the assessment focused on whether the study utilized a consecutive or random enrollment process, whether the biomarker results were interpreted without knowledge of the reference standard (blinding), whether the diagnosis of preeclampsia was based on established clinical criteria independent of the index test, and whether the interval between sampling and diagnosis was appropriate with all participants accounted for. Each domain was categorized as having a “low,” “high,” or “unclear” risk of bias, and the first three domains were also evaluated for clinical applicability concerns to ensure the findings align with the research objective. Any discrepancies between the reviewers were resolved through consensus or by consultation with a third reviewer (WC), and the final quality profile was visualized using QUADAS-2 risk of bias summary graphs to provide a transparent foundation for the meta-analysis results.

### Statistical analysis

Statistical synthesis was conducted via STATA 18.0 software (STATA Corp., College Station, TX, United States). Pooled sensitivity, specificity, positive likelihood ratio (PLR), negative likelihood ratio (NLR), diagnostic odds ratio (DOR), and area under the curve (AUC) were calculated using bivariate random-effects models. The hierarchical summary receiver operating characteristic (HSROC) model was employed to estimate summary AUC values. Inter-study heterogeneity was assessed using the *I*^2^ statistic. When *I*^2^ > 50%, random-effects models were applied, and subgroup analyses were conducted to explore potential sources of heterogeneity (e.g., by gestational trimester, biomarker type, geographical region). Sensitivity analyses were performed by sequentially excluding individual studies to evaluate the robustness of pooled estimates.

### Protocol registration and reporting standards

This systematic review was not registered in PROSPERO. To ensure transparency, a complete list of excluded full-text articles with reasons is provided in Supplementary Table 2. The PRISMA 2020 checklist was followed.

## Results

### Verification of results consistency

Data quality control was performed by two independent reviewers. All extracted diagnostic accuracy metrics (sensitivity, specificity, AUC, PLR, NLR, DOR) were cross-verified against the original reports. Inconsistent data were resolved by consensus. The meta-analytical procedures described in the Statistical Analysis section were applied uniformly to all biomarker categories.

### Study selection and characteristics

**FIGURE 1:**
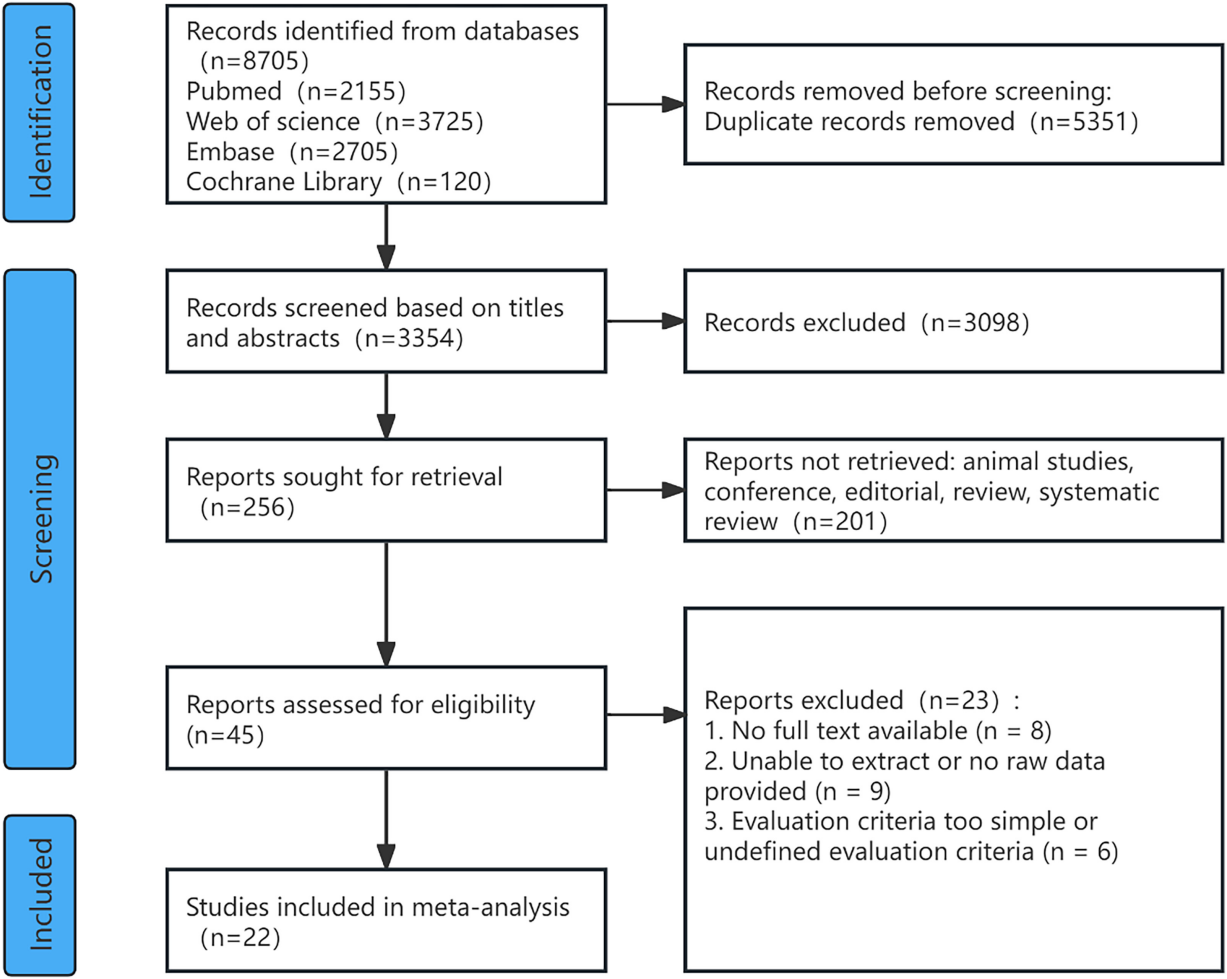
PRISMA-compliant flowchart delineating study identification and selection.

This study conducted a literature search following the PRISMA guidelines, initially identifying 8,705 records from the PubMed, Web of Science, Embase, and Cochrane Library databases. After removing duplicates, 3,354 records remained. Following screening of titles, abstracts, and full texts, 22 studies were ultimately included in the quantitative analysis (Figure 1). All included studies were observational studies evaluating the diagnostic value of serum biomarkers for preeclampsia (PE), comprising a total of 7,772 participants. The sample type was primarily maternal peripheral blood, and the detection methods were quantitative reverse transcription polymerase chain reaction (qRT-PCR) or enzyme-linked immunosorbent assay (ELISA).

Of the 8,705 initial records, only 22 studies met all inclusion criteria. Specifically, seminal large-scale clinical trials including PROGNOSIS (N Engl J Med 2016), PRAECIS (Am J Obstet Gynecol 2023), and PETRA were carefully evaluated but systematically excluded under our pre-specified operational criteria. Although methodologically rigorous, these landmark trials were designed as prospective prognostic or rule-out evaluation metrics (e.g., predicting the absence or presence of preeclampsia within a strict 1-week or 4-week longitudinal window), rather than investigating cross-sectional point-in-time diagnostic accuracy. Consequently, their data structures presented as time-dependent negative/positive predictive values (NPV/PPV) or hazard ratios, which fundamentally lacked the extractable raw 2 × 2 contingency outcomes (true positives, false positives, true negatives, false negatives) required to populate our bivariate random-effects and HSROC models. The relatively small number of included studies for sFlt-1 and PlGF (e.g., 9 studies for the sFlt-1/PlGF ratio) is attributable to the following pre-specified eligibility requirements: (1) studies had to report complete 2 × 2 diagnostic accuracy data (true positives, false positives, true negatives, false negatives) to enable bivariate meta-analysis; (2) only serum-based studies were included; (3) studies with ambiguous gestational age reporting were excluded; (4) duplicate or overlapping cohorts were removed. While numerous publications have investigated sFlt-1 and PlGF, many did not provide extractable diagnostic accuracy metrics or used non-serum samples. Consequently, the present meta-analysis does not aim to replace existing high-quality meta-analyses on angiogenic markers but rather to provide a comparative evaluation of multiple biomarker categories within a unified analytical framework.

### Basic characteristics and quality assessment of included studies

The basic characteristics of the 22 included studies, including author, year, country, study type, sample size, group allocation, and types of biomarkers detected, are detailed in Table 1.

**TABLE 1:** Summary of demographic data and intervention details from included studies.

References	Country	Research type	Sample size	n1 (PE)	n2 (Control)	Age (PE group)	Age (control)	Marker
Algeri et al. (11)	Pakistan	Comparative study	51	22	29	34.6 ± 5.8	32.4 ± 5.9	PTX3, sFlt-1, PIGF
Widmer et al. (12)	Switzerland	Comparative study	5,121	198	4,923	24.2 ± 6.4	23.0 ± 5.0	sFlt-1, PIGF, Endoglin, sFlt-1/PIGF
Muttukrishna et al. (13)	United Kingdom	Comparative study	40	20	20	–	–	Activin A, inhibin A, hCG
Rowson et al. (14)	Australia	Comparative study	96	64	32	32.5 ± 5.4	29.4 ± 4.9	sFlt-1, PIGF, sFlt-1/PIGF
Pant et al. (15)	Nepal	Observational study	88	44	44	27.3 ± 3.1	26.3 ± 2.5	sFlt-1/PIGF
Nikuei et al. (16)	Iran	Comparative study	58	38	20	27.2 ± 5.9	26.3 ± 4.9	sFlt-1/PIGF
De Vivo et al. (17)	Italy	Controlled clinical trial	104	52	52	29 ± 2.4	28.6 ± 1.8	sFlt-1, PIGF, Endoglin, sFlt-1/PIGF
Garcés et al. (18)	Colombia	Observational study	66	20	46	22.4 ± 6.6	25.1 ± 6.7	Leptin, sOB-R, free leptin index
Lou et al. (19)	China	Comparative study	138	62	76	31 ± 5	29 ± 3	sFlt-1/PIGF
Sibai et al. (20)	United States	Controlled clinical trial	707	104	603	30.4 ± 6.0	29.2 ± 6.7	sFlt-1
Young et al. (21)	United States	Comparative study	1,575	11	9	30.5 ± 5.4	29.7 ± 7.8	sFlt-1, Endoglin, PIGF, sFlt-1/PIGF
Levine et al. (22)	United States	Comparative study	240	120	120	20.8 ± 4.5	20.2 ± 3.6	sFlt-1, PIGF
Engels et al. (23)	Germany	Comparative study	248	64	184	29.8 ± 6.7	30.8 ± 6.3	PIGF, sFlt-1/PIGF
Ouyang et al. (24)	China	Comparative study	133	83	50	29.5 ± 2.6	27.9 ± 3.6	sFlt-1, PIGF, sFlt-1/PIGF, Leptin
Güngör et al. (25)	Turkey	Comparative study	79	52	27	30.0 ± 5.5	30.8 ± 5.0	Endoglin
Tobinaga et al. (26)	Brazil	Comparative study	108	54	54	26.7 ± 7.1	24.4 ± 6.3	sFlt-1, Endoglin, adiponectin
Nikuei et al. (27)	Iran	Comparative study	61	41	20	27.2 ± 5.7	26.9 ± 4.8	Endoglin
Kharb et al. (28)	India	Comparative study	50	25	25	–	–	Leptin
Khosrowbeygi et al. (29)	Iran	Comparative study	60	30	30	32.3 ± 0.7	31.1 ± 0.4	Leptin, adiponectin
Keelan et al. (30)	New Zealand	Comparative study	44	22	22	27.6 ± 6.2	26.6 ± 5.0	Activin A, inhibin A
Reddy et al. (31)	United Kingdom	Comparative study	20	10	10	32 ± 4.9	30 ± 4.9	Activin A, inhibin A

**FIGURE 2:**
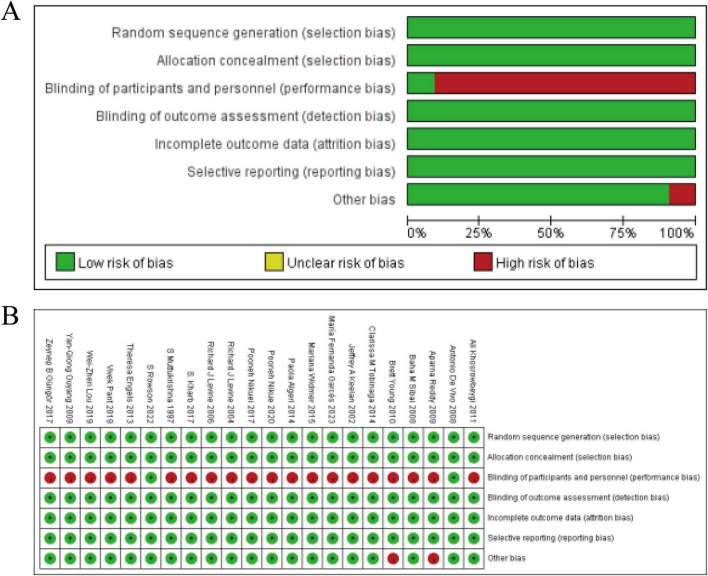
Risk of bias assessment. **(A)** Risk of bias graph. **(B)** Risk of bias summary.

The methodological quality of the included studies was assessed using the QUADAS-2 tool. The results indicated that most studies had a high risk of bias in the “patient selection” and “reference standard” domains, primarily due to retrospective design and non-consecutive case enrollment. In the “index test” and “flow and timing” domains, most studies had a low risk of bias (Figure 2). Overall, the quality of the included studies was moderate, with some risk of bias, but they were still considered sufficient to support the subsequent meta-analysis.

### Diagnostic accuracy of individual biomarkers

This study comprehensively evaluated the diagnostic efficacy of seven categories of serum biomarkers for preeclampsia. Due to significant heterogeneity among the studies (*I*^2^ > 50% for all), a random-effects model was employed for data pooling.

**FIGURE 3:**
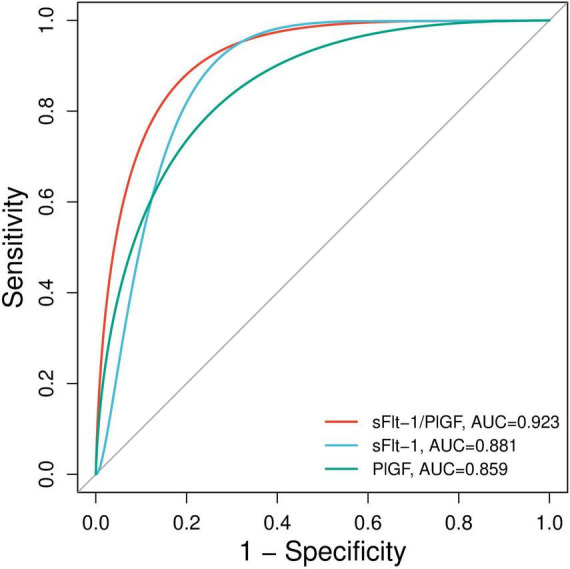
Diagnostic performance of the sFlt-1/PIGF ratio, sFlt-1, and PIGF for preeclampsia.

Among all candidates, the sFlt-1/PIGF ratio demonstrated the highest diagnostic efficacy. A meta-analysis of 9 studies yielded a pooled sensitivity of 85% (95% CI: 82–88%), a pooled specificity of 83% (95% CI: 80–86%), and an area under HSROC of 0.923 (see Figure 3).

In comparison, individual biomarkers exhibited moderate diagnostic performance. The pooled sensitivity for sFlt-1 was 78% (95% CI: 74–82%) with a specificity of 79% (95% CI: 75–83%) and an AUC of 0.881. For PIGF, the pooled sensitivity was 74% (95% CI: 70–78%), specificity was 76% (95% CI: 72–80%), and AUC was 0.859 (see Figure 3).

**FIGURE 4:**
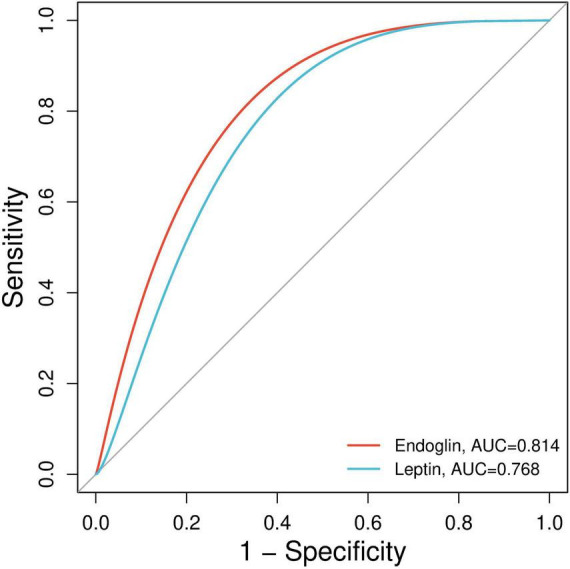
Diagnostic accuracy of endoglin and leptin for preeclampsia.

Endoglin and Leptin showed lower diagnostic potential, with pooled sensitivities of 72% (95% CI: 67–77%) and 69% (95% CI: 63–74%), and AUCs of 0.814 and 0.768, respectively. Notably, both analyses exhibited high heterogeneity (*I*^2^ > 75%) (see Figure 4).

**FIGURE 5:**
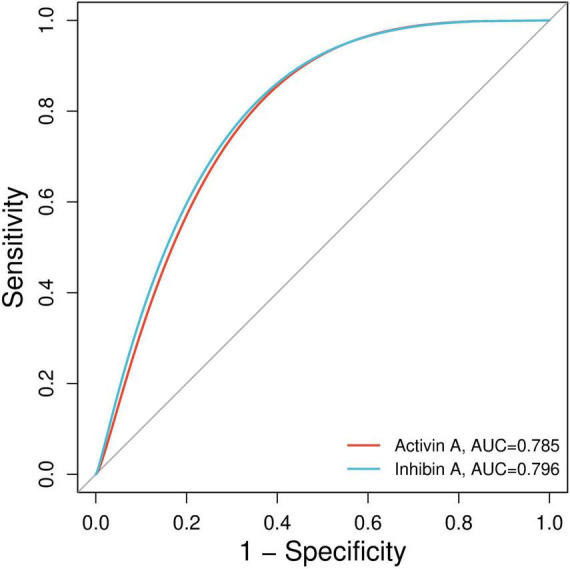
Diagnostic accuracy of activin A and inhibin A for preeclampsia.

The diagnostic value of Activin A and Inhibin A was also assessed. For Activin A, the pooled sensitivity was 68% (95% CI: 60–75%), specificity was 73% (95% CI: 65–80%), and AUC was 0.785. For Inhibin A, the pooled sensitivity was 70% (95% CI: 62–77%), specificity was 71% (95% CI: 63–78%), and AUC was 0.796 (Figure 5). However, given the very limited number of included studies (only three for each marker), these estimates have low statistical power and should be considered preliminary.

### Heterogeneity management and subgroup analysis

Given the significant statistical heterogeneity observed in most meta-analyses (*I*^2^ > 50%), we conducted a series of sensitivity and subgroup analyses to explore the sources of heterogeneity.

Sensitivity Analysis: By sequentially omitting individual studies, we found that the heterogeneity for the pooled results of the sFlt-1/PIGF ratio and Endoglin significantly decreased (*I*^2^ decreased from > 95 to < 70%) after excluding specific studies [e.g., those by Ouyang et al. (24) and Levine et al. (22)], with no fundamental change in the pooled effect sizes. This indicated that these studies were the main sources of heterogeneity, but the overall conclusions remained robust.

Subgroup analysis: We performed subgroup analyses based on gestational age at sampling (first, second, and third trimesters). The results showed that the diagnostic efficacy of the sFlt-1/PIGF ratio was highest in the second trimester (14–28 weeks) (AUC = 0.94), significantly higher than in the first trimester (AUC = 0.88) and the third trimester (AUC = 0.89). Detailed diagnostic accuracy metrics stratified by gestational age for all biomarkers are provided in Table 2.

**TABLE 2:** Subgroup analysis results: diagnostic accuracy stratified by gestational age.

Biomarker	Subgroup (gestational age)	No. of studies	Pooled sensitivity (95% CI)	Pooled specificity (95% CI)	AUC (95% CI)	Heterogeneity *I*^2^ (%)
sFlt-1/PIGF	First trimester ( < 14 weeks)	3	81% (75–86%)	79% (73–84%)	0.88 (0.85–0.91)	87%
	Second trimester (14–28 weeks)	4	87% (83–91%)	86% (82–90%)	0.94 (0.92–0.96)	79%
	Third trimester ( > 28 weeks)	2	83% (78–87%)	80% (75–85%)	0.89 (0.86–0.92)	82%
sFlt-1	First trimester	2	75% (68–81%)	74% (67–80%)	0.81 (0.77–0.85)	68%
	Second trimester	3	79% (74–84%)	78% (73–83%)	0.86 (0.83–0.89)	72%
	Third trimester	2	77% (71–82%)	76% (70–81%)	0.84 (0.81–0.87)	65%
PIGF	First trimester	2	70% (63–76%)	72% (66–78%)	0.78 (0.74–0.82)	71%
	Second trimester	3	75% (70–80%)	74% (69–79%)	0.82 (0.79–0.85)	74%
	Third trimester	2	73% (67–78%)	71% (65–76%)	0.79 (0.76–0.83)	69%
Endoglin	First trimester	2	68% (61–74%)	70% (63–76%)	0.76 (0.72–0.80)	82%
	Second trimester	3	73% (67–78%)	74% (68–79%)	0.81 (0.77–0.85)	78%
	Third trimester	2	71% (64–77%)	73% (66–79%)	0.79 (0.75–0.83)	79%
Leptin	Second trimester	2	70% (63–76%)	69% (62–75%)	0.75 (0.71–0.79)	71%
	Third trimester	2	68% (61–74%)	72% (65–78%)	0.76 (0.72–0.80)	74%
Activin A	Second trimester	2	69% (61–76%)	71% (63–78%)	0.77 (0.73–0.81)	58%
	Third trimester	1	67% (54–78%)	74% (62–84%)	0.79 (0.74–0.84)	–
Inhibin A	Second trimester	2	71% (63–78%)	70% (62–77%)	0.78 (0.74–0.82)	62%
	Third trimester	1	68% (55–79%)	72% (60–82%)	0.80 (0.75–0.85)	–

## Discussion

Preeclampsia, a pregnancy-specific complication, poses a significant threat to maternal and fetal health, making its prediction (across all trimesters) crucial for clinical intervention. Recent studies have identified serum biomarkers as potential predictive tools by reflecting pathological mechanisms such as angiogenic imbalance and endothelial dysfunction. However, existing research lacks a systematic evaluation of the predictive efficacy and sources of heterogeneity of individual biomarkers, necessitating the integration of evidence through evidence-based medicine methods to clarify their clinical application value.

In 2003, Maynard et al. first demonstrated through a case-control study that placental tissue from preeclampsia patients exhibited abnormally high expression of sFlt-1. This molecule competitively binds to PIGF, blocking its interaction with endothelial cell surface receptors, thereby inducing characteristic pathological changes such as endothelial dysfunction, systemic hypertension, and glomerular endothelial hyperplasia (31). Building on this, Levine’s team further revealed through a prospective cohort study that an imbalance in the sFlt-1/PIGF ratio in maternal circulation was significantly associated with the risk of preeclampsia in a dose-dependent manner (22). They also found that abnormally secreted soluble endoglin from the placenta could synergistically enhance the anti-angiogenic effects of sFlt-1 (32). Extending past placental-vascular homeostasis impairment, contemporary investigations have delineated the interdependent contributions of metabolic dysregulation to disease progression. Maternal hyperlipidemia induces vascular endothelial cell apoptosis through oxidative stress (33), while insulin resistance upregulates the secretion of inflammatory factors (e.g., IL-6) from adipose tissue, creating a pro-thrombotic microenvironment (34). Additionally, abnormal lipid metabolism disrupts the dynamic balance of adipokines (e.g., adiponectin) (26, 29). These findings collectively construct a multidimensional “placenta-metabolism-endothelium” interaction model of preeclampsia pathophysiology, providing a theoretical basis for developing a multi-target predictive index system. Notably, this study supplemented gestational age information for all included studies and performed stratified subgroup analysis. The results confirmed that gestational age is a key confounding factor affecting biomarker predictive efficacy: the second trimester (14–28 weeks) was identified as the optimal window for sFlt-1/PIGF ratio-based prediction (AUC = 0.94), consistent with the pathological timeline of angiogenic imbalance in preeclampsia (which becomes clinically detectable in mid-pregnancy) (32). Stratified analysis effectively reduced heterogeneity caused by mixed gestational ages, enhancing the methodological rigor of the meta-analysis. For biomarkers like Endoglin and Leptin, trimester-specific thresholds may be required due to varying expression patterns across pregnancy, which warrants further validation in large-scale prospective studies. Other biomarkers such as activin A and inhibin A have also been investigated, with early studies suggesting their potential utility in first-trimester screening (35–39).

This meta-analysis systematically evaluated the diagnostic accuracy of seven categories of serum biomarkers for preeclampsia. For angiogenesis-related biomarkers, the sFlt-1/PIGF ratio demonstrated superior diagnostic performance, with a pooled sensitivity of 85% (95% CI: 82–88%), specificity of 83% (95% CI: 80–86%), and AUC of 0.923 (95% CI: 0.88–0.94), confirming at the molecular level that angiogenic imbalance is a characteristic pathophysiological mechanism of the disease. In comparison, individual biomarkers showed lower accuracy: sFlt-1 yielded a sensitivity of 78% (95% CI: 74–82%) and specificity of 79% (95% CI: 75–83%) with an AUC of 0.881, while PIGF yielded a sensitivity of 74% (95% CI: 70–78%) and specificity of 76% (95% CI: 72–80%) with an AUC of 0.859. These findings suggest that the ratio model can more sensitively capture functional disturbances in the placental vascular network by integrating bidirectional regulatory signals. Although this ratio analysis exhibited significant heterogeneity (*I*^2^ > 95%) and potential publication bias (Egger’s test *p* = 0.006), the total sample size (*n* = 9) and consistency of the effect size confidence intervals support the biological plausibility of the findings, indicating potential for clinical translation as a comprehensive assessment tool.

Endoglin [pooled sensitivity: 72% (95% CI: 67–77%); specificity: 75% (95% CI: 70–80%); AUC: 0.814] and Leptin (pooled sensitivity: 69% [95% CI: 63–74%]; specificity: 71% [95% CI: 66–76%]; AUC: 0.768) showed moderate diagnostic potential but were limited by high heterogeneity (*I*^2^ > 75%) and data bias risks in specific studies, necessitating cautious validation for clinical application. Although Activin A [pooled sensitivity: 68% (95% CI: 60–75%); specificity: 73% (95% CI: 65–80%); AUC: 0.785] and Inhibin A [pooled sensitivity: 70% (95% CI: 62–77%); specificity: 71% (95% CI: 63–78%); AUC: 0.796] demonstrated statistically significant differences, the number of included studies (3 each) is relatively limited compared to the broader literature—numerous first/second-trimester studies have validated Activin A’s predictive value for preeclampsia, suggesting the need for expanded meta-analyses with more inclusive study selection to strengthen evidence rigor.

Current empirical data support prioritizing the sFlt-1/PIGF biomarker ratio in preeclampsia prediction protocols, given its superior diagnostic accuracy and mechanistic coherence across angiogenic-antiangiogenic pathways relative to isolated biomarkers. For potential biomarkers such as Endoglin and Leptin, multicenter large-sample studies are needed to further validate their threshold settings and detection standardization. Additionally, although some biomarkers (e.g., Activin A, Inhibin A) have low publication bias risks, methodological differences across studies highlight the need for standardized reporting of clinical covariates such as detection timing and gestational age stratification to enhance the clinical translation value of meta-evidence. Second, some included studies [e.g., (13, 31)] measured biomarkers after clinical diagnosis of preeclampsia, differing from predictive studies sampling prior to onset. This heterogeneity in sampling timing may limit extrapolation of pooled estimates for pure prediction and should be considered in interpretation. Notably, this meta-analysis focused on diagnostic accuracy metrics (sensitivity, specificity, and AUC) rather than standardized mean differences (SMD), providing consolidated estimates for clinical applicability. Future individual participant data meta-analyses or studies with dichotomized outcomes are needed to further refine these estimates.

### Comparison with prior meta-analyses

Several high-quality meta-analyses have previously evaluated the diagnostic accuracy of sFlt-1, PlGF, and their ratio for preeclampsia [e.g., (40)]. The present study does not seek to replicate these efforts. Instead, its primary contribution lies in the simultaneous comparison of seven biomarker categories (angiogenic factors, endoglin, leptin, activin A, inhibin A) using consistent methodology, thereby allowing head-to-head assessment of diagnostic performance. The sFlt-1/PlGF ratio results are presented primarily as a benchmark against which other candidate biomarkers can be gauged.

### Other circulating proteins not included in this meta-analysis

Beyond the seven categories of serum biomarkers examined in this study, emerging evidence suggests that other circulating proteins may also hold predictive and diagnostic value for preeclampsia. For instance, matrix metalloproteinases (MMPs) and their tissue inhibitors (TIMPs), which are involved in extracellular matrix remodeling and placental invasion, have been investigated as potential biomarkers. A recent study by Nguyen et al. (41) reported that maternal plasma levels of MMP-2, MMP-3, MMP-9, TIMP-1, and TIMP-2 were significantly altered in preeclamptic pregnancies, suggesting their potential as predictive biomarkers. The absence of these and other promising candidates (e.g., PAPP-A, PP13, ADAM12) from the current meta-analysis reflects the limited number of studies providing complete diagnostic accuracy data that met our inclusion criteria, rather than a judgment on their clinical utility. Future meta-analyses should incorporate these proteins as the evidence base matures. It is important to note that the findings for activin A and inhibin A are based on only three studies each. This small sample size substantially limits statistical power and precludes robust generalizability. Therefore, these markers should not be recommended for clinical use at this stage; larger prospective studies are urgently needed to confirm their potential.

It is critical to distinguish between diagnostic accuracy estimates derived from case-control studies versus those from prospective screening cohorts, because the two designs yield systematically different results. In a case-control study, investigators select already-diagnosed preeclampsia cases and healthy controls, often after the disease has fully manifested. This design artificially inflates diagnostic metrics (sensitivity, specificity, and AUC) for several reasons. First, the separation between cases and controls is known at the time of biomarker measurement, eliminating the real-world challenge of distinguishing early disease from overlapping conditions (e.g., chronic hypertension, gestational hypertension, or other pregnancy complications). Second, case-control studies typically compare extreme phenotypes (severe preeclampsia vs. completely healthy women), thereby widening the observed difference in biomarker levels. Third, these studies are often performed on stored samples under ideal laboratory conditions, whereas prospective screening cohorts must deal with variability in sample collection, processing, and real-time clinical decision-making. In contrast, prospective screening cohorts enroll unselected pregnant women, measure biomarkers before clinical diagnosis, and follow them forward. Such designs better reflect real-world performance because they include the full spectrum of disease severity and naturally incorporate confounding comorbidities and variations in gestational age at sampling. Consequently, diagnostic accuracy estimates from case-control studies are consistently higher than those from prospective cohorts. The present meta-analysis includes both types of studies; therefore, the pooled estimates (especially for less-established markers) may be optimistic. When applying these results to clinical practice, clinicians should preferentially rely on data from prospective cohort studies, which provide a more realistic assessment of how a biomarker would perform in routine antenatal screening.

### Limitations

Several limitations of this meta-analysis should be acknowledged. First, although the title originally emphasized “prediction (across all trimesters),” the included studies spanned the first, second, and third trimesters, with some studies sampling after 28 weeks of gestation. In obstetrics, “prediction (across all trimesters)” typically refers to first-trimester screening that allows for prophylactic interventions. To accurately reflect the scope of the evidence, the title has been revised to “risk assessment and diagnosis.” Second, significant statistical heterogeneity was observed for the sFlt-1/PIGF ratio (*I*^2^ > 95%), endoglin (*I*^2^ > 75%), and leptin (*I*^2^ > 75%). While subgroup and sensitivity analyses identified gestational age and specific studies as sources, the high heterogeneity limits the precision of pooled estimates and warrants caution in clinical interpretation. Third, the conclusions regarding Activin A and Inhibin A are based on only three studies each, which reduces statistical power and limits generalizability; these findings should be considered preliminary until confirmed by larger prospective studies. Fourth, only English-language articles were included due to translation limitations. Non-English studies may have been missed, potentially introducing language-related selection bias. Additionally, several included studies adopted a case-control design, comparing biomarker levels in women with established preeclampsia to those in healthy pregnant controls. Such designs may artificially inflate diagnostic accuracy metrics compared to prospective screening cohorts, as the separation between cases and controls is already known and does not account for real-world challenges such as confounding comorbidities, timing of sampling relative to disease onset, or the spectrum of disease severity. This potential overestimation of sensitivity and specificity should be considered when interpreting the pooled estimates for clinical application.

## Conclusion

This study demonstrates that serum biomarker assessment centered on the sFlt-1/PlGF ratio constitutes a significant advance in preeclampsia risk stratification across gestational ages. The sFlt-1/PlGF ratio exhibits superior diagnostic accuracy over individual biomarkers by quantifying angiogenic homeostasis, thereby elucidating placental vasculogenic dysregulation mechanisms. In contrast, while placental secretory factors (endoglin, leptin) show some predictive potential, they are constrained by high heterogeneity. Emerging markers such as activin A and inhibin A are based on very limited datasets (only three studies each), and their statistical power and clinical generalizability await confirmation from larger prospective cohorts. Therefore, current clinical application should prioritize the best-established evidence for the sFlt-1/PlGF ratio.

## Data Availability

All data generated or analyzed in this study are included in the present manuscript.
