# Supplementary material for: Serum biomarkers for the prediction and diagnosis of preeclampsia: a systematic meta-analysis of diagnostic accuracy
Source: Front Med (Lausanne). 2026 Aug 3;13:1861345. doi: 10.3389/fmed.2026.1861345 (PMC13478171; doi:10.3389/fmed.2026.1861345)
Supplement: Supplementary file 1 [file Table_1.docx]

**Supplementary Table 1. Search strategies for each database**

| Database | Search Strategy (including filters) | Results |
| --- | --- | --- |
| PubMed | ("preeclampsia"[MeSH Terms] OR "pre-eclampsia"[Title/Abstract] OR "hypertensive disorders of pregnancy"[Title/Abstract]) AND ("biomarkers"[MeSH Terms] OR "serum marker"[Title/Abstract] OR "sFlt-1"[Title/Abstract] OR "PlGF"[Title/Abstract] OR "PIGF"[Title/Abstract] OR "endoglin"[Title/Abstract] OR "leptin"[Title/Abstract] OR "activin A"[Title/Abstract] OR "inhibin A"[Title/Abstract]) AND ("diagnosis"[MeSH Terms] OR "prediction"[Title/Abstract] OR "screening"[Title/Abstract]) Filters: Humans, English, Journal Article; from inception to 2026/01/20 | 2,103 |
| Embase | ('preeclampsia'/exp OR 'pre-eclampsia':ab,ti OR 'hypertensive disorder of pregnancy':ab,ti) AND ('biomarker'/exp OR 'serum marker':ab,ti OR 'sFlt-1':ab,ti OR 'PlGF':ab,ti OR 'PIGF':ab,ti OR 'endoglin':ab,ti OR 'leptin':ab,ti OR 'activin A':ab,ti OR 'inhibin A':ab,ti) AND ('diagnosis'/exp OR 'prediction':ab,ti OR 'screening':ab,ti) Filters: (human)/lim, (english)/lim, article; from 1974 to 2026/01/20 | 3,247 |
| Web of Science | TS=(preeclampsia OR "pre-eclampsia" OR "hypertensive disorders of pregnancy") AND TS=(biomarker* OR "serum marker*" OR sFlt-1 OR PlGF OR PIGF OR endoglin OR leptin OR "activin A" OR "inhibin A") AND TS=(diagnosis OR prediction OR screening) Filters: English, Article; from inception to 2026/01/20 | 2,689 |
| Cochrane Library | (preeclampsia OR pre-eclampsia OR "hypertensive disorders of pregnancy") AND (biomarker* OR "serum marker*" OR sFlt-1 OR PlGF OR PIGF OR endoglin OR leptin OR "activin A" OR "inhibin A") AND (diagnosis OR prediction OR screening) Filters: Trials, Reviews (no language restriction due to small yield); from inception to 2026/01/20 | 666 |
