# Supplementary material for: Serum biomarkers for the prediction and diagnosis of preeclampsia: a systematic meta-analysis of diagnostic accuracy
Source: Front Med (Lausanne). 2026 Aug 3;13:1861345. doi: 10.3389/fmed.2026.1861345 (PMC13478171; doi:10.3389/fmed.2026.1861345)
Supplement: Supplementary file 2 [file Table_2.docx]

**Supplementary Table 2. List of excluded full‑text articles with reasons for exclusion**

| **Excluded study (first author, year / trial name)** | **Reason for exclusion** |
| --- | --- |
| PROGNOSIS study group (N Engl J Med 2016) | Lacked extractable 2×2 diagnostic contingency data; evaluated longitudinal short‑term prediction/prognosis (1‑week/4‑week rule‑out) rather than cross‑sectional diagnostic accuracy. |
| PRAECIS study group (Am J Obstet Gynecol 2023) | Same as above: designed for prognostic/rule‑out evaluation; no 2×2 data for bivariate meta‑analysis. |
| PETRA study group (NCT02051387) | Focused on short‑term prediction (delivery within 4 weeks); outcome definition not aligned with diagnostic accuracy; no extractable 2×2 data. |
| Zhang Y, et al., 2019 (Chinese study) | Non‑English full‑text; no translation available. |
| López M, et al., 2018 (Spanish study) | Non‑English full‑text; no translation available. |
| Schmidt A, et al., 2020 (German study) | Non‑English full‑text; no translation available. |
| Ivanova E, et al., 2017 (Russian study) | Non‑English full‑text; no translation available. |
| Chen H, et al., 2015 | Duplicate publication of Ouyang et al., 2009; same patient cohort. |
| Park SY, et al., 2016 | Case‑control with biomarker measurement after clinical diagnosis; no prospective sampling; potential overestimation of accuracy. |
| Kaur M, et al., 2014 | Conference abstract only; full‑text not available; insufficient data for meta‑analysis. |
| Morales‑Roselló J, et al., 2019 | Review article, not an original diagnostic accuracy study. |
| Wang L, et al., 2021 | No serum biomarker data; studied urinary biomarkers only. |
| Al‑Rubaie Z, et al., 2018 | Incomplete outcome data; sensitivity and specificity not reported and could not be calculated from available information. |
